# The Tool for Automatic Analysis of Decoding Ambiguity (TAADA)

**DOI:** 10.3758/s13428-025-02922-w

**Published:** 2026-01-16

**Authors:** Scott Crossley, Joon Suh Choi, Kenny Tang, Laurie Cutting

**Affiliations:** 2301 Vanderbilt Place, Nashville, TN 37240 USA

**Keywords:** Natural language processing, Text decoding, Text readability, Lexical processing

## Abstract

This study documents and assesses the Tool for Automatic Analysis of Decoding Ambiguity (TAADA). TAADA calculates measures related to decoding, including metrics for grapheme and phoneme counts, neighborhood effects, rhymes, and conditional probabilities for sound–spelling relationships. These measures are assessed in two reading studies. The first study examined links between decoding variables and judgments of reading ease in a corpus of ~5000 reading excerpts, finding that variables related to word frequency, phonographic neighbors for words, word syllable length, and the reverse prior probability for consonants explained 34% of the variance in the reading scores. The second examined links between decoding variables and student reading miscues, finding that word frequency, phoneme counts, rhyme counts, and probability counts explained 3% of students’ reading miscues.

## Introduction

The ability to read is a critical skill that is associated with positive academic and social outcomes throughout a person’s lifetime (Kern & Friedman, 2009; McLaughlin et al., 2014). However, in the United States, national reading assessments demonstrate that around 70% of students are not reading at grade-level (U.S. Department of Education, 2024). An important component of transitioning these students to grade-level proficiency is to ensure that they are exposed to texts that match their reading level so that their reading activities complement their skills and can lead to increased reading proficiency (Cheatham & Allor, 2012; Allington, 2005; Stanovich, 1985; Wolfe et al., 1998).

One way to match readers to texts is to quantify language features within texts to examine links between comprehension and text complexity. For instance, research indicates that a text will likely be more difficult to read if it contains more sophisticated words that are difficult to recognize, more complex sentence structures, and fewer cohesive features that bind sentences and paragraphs together (Just & Carpenter, 1980; Snow, 2002). Text complexity factors work in conjunction with reader and task factors to explain text readability. Indeed, research indicates that text comprehension is best explained by readers’ background knowledge or reading ability (O’Reilly et al., 2019; Wang et al., 2017) and that comprehension is also related to genre, with narrative texts being easier to process than informative or expository texts (Best, Floyd, & McNamara, 2008; Saenz & Fuchs, 2002).

In terms of lexical sophistication, studies have shown that English word properties including imageability, concreteness (Coltheart et al., 1988; Crossley et al., 2023; Pickren, Stacy, Del Tufo, Spencer & Cutting, 2022; Richardson, 1975), word familiarity, age of exposure (Coltheart et al., 1988; Crossley et al., 2023; Howes & Solomon, 1951), word association strength (Crossley et al., 2023), and semantics (Collins-Thompson, 2014; Mesmer et al., 2012; Seidenberg et al., 2022) all influence word recognition and text comprehension. For English words, lexical features related to frequency are strong predictors of text processing (Adelman et al., 2013; Crossley et al., 2017; Taylor & Perfetti, 2016) and text comprehension (Crossley et al., 2023). At the sub-lexical level, features related to sound and spelling relationships between words (Ehri et al., 2001; Juel & Solso, 1981; Mesmer, 2005), the number of blends in a word, discrepancies between the number of letters and phonemes in a word (Saha et al., 2021), rhyme counts, and neighborhood effects (Crossley & Choi, 2024) are all related to reading comprehension. Features related to the number of graphemes in a word and word neighborhood effects have also shown significant relationships with judgments of text processing difficulty (Crossley et al., 2017; Taylor & Perfetti, 2016). Recent research has quantified some of these lexical and sub-lexical features into an automated measure of *decoding difficulty* (Saha et al., 2021), which was found to be predictive of miscues when reading passages. While this measure includes metrics of word frequency, its primary focus is to capture the complexity of phonological–orthographic relationships, or how difficult a word is to sound out. The motivation for this measure was a need for a metric to capture decoding difficulty in texts. Such a metric is particularly needed for selecting and developing texts that are aligned with instruction on phonological-orthographic mappings, which is a heavily emphasized component of reading instruction for beginning readers and those with dyslexia. However, additional research is needed to provide and assess more robust, thorough, and reliable metrics of decoding difficulty in English.

The goal of this paper is to document and assess the Tool for Automatic Analysis of Decoding Ambiguity (TAADA). TAADA was developed to annotate and count lexical and sub-lexical features related to reading words in English (generally for standard American English). The counts provided in TAADA include lexical features related to word frequency and contextual diversity along with sub-lexical features including metrics for grapheme and phoneme counts, neighborhood effects, rhymes, and conditional probabilities for sound–spelling relationships. We assess the features reported in TAADA in two reading studies. The first study investigates more holistic relationships between the TAADA variables and human judgements of reading comprehension in a corpus of ~5000 reading excerpts. This study tests the hypothesis that excerpts containing words that are more difficult to decode are harder to comprehend. The second study is a more fine-grained analysis that examines relationships between the variables in TAADA and reading miscue rates for children during reading. This study tests the hypothesis that lexical and sub-lexical features of words predict the number of miscues while reading aloud.

## Word recognition, decoding, and reading

Word recognition refers to the ability to recognize both spoken and written words (Jenkins et al., 2003; Just & Carpenter, 1987; Perfetti, 1985). While some words may be recognized automatically due to the frequency or the quality of a lexical mapping of that word, unfamiliar words typically need to be decoded by applying knowledge of phonological-orthographic mappings. According to the simple view of reading (SVR), decoding, along with linguistic comprehension, are two key skills that together predict individual’s ability to understand written text (Gough et al., 1996; Gough & Tunmer, 1986; Hoover & Gough, 1990). Gough and colleagues argued that decoding relies on knowing the spelling-sound correspondences in English and is a necessary component of reading success. Evidence linking decoding and reading comprehension is generally strong, with some studies reporting that decoding measures can explain upward of 90% of the variance in reading comprehension (Katzir et al., 2006). However, Katzir et al. acknowledged that there was not a one-to-one correspondence between graphemes and phonemes and that good readers may recognize words in the absence of decoding them (Gough & Tunmer, 1986). Indeed, some studies have found that decoding is a negligible predictor of comprehension (e.g., Berninger et al., 2006).

While the SVR is one of the most widely adopted developmental models of reading, other theoretical models offer more in-depth hypotheses about how word-specific representations influence reading outcomes (Nation et al., 2007; Ehri, 2014; Perfetti & Stafura, 2014). More specifically, according to the Lexical Quality Hypothesis (LQH; Perfetti & Hart, 2008), skilled readers have word representations that are well specified for orthographic, phonological, and semantic-syntactic information, which results in efficient word recognition. Phonological-orthographic representations are typically not strong in beginning readers and those with dyslexia. However, the LQH posits that word representations on all three dimensions become solidified as children become more proficient readers, thus facilitating fast and efficient word recognition.

While the SVR components and robustness of lexical representations have been found to be central for reading comprehension, research has demonstrated that there are other linguistically-related predictors of reading, as well as moderators of the decoding–linguistic comprehension relationship. For example, after controlling for decoding and listening comprehension, other predictors of reading comprehension include vocabulary knowledge (Savage & Stewart, 2006; Tilstra et al., 2009), reading fluency (Eason et al., 2013; Tilstra et al., 2009), and naming speed (Johnston & Kirby, 2006). In terms of moderators relevant to the SVR, a meta-analysis by Garcia and Cain (2014) found that the decoding–reading comprehension relationship decreased with increasing age and that characteristics of the reading assessment influenced comprehension. These characteristics include genre (narrative texts lead to greater comprehension than expository texts), whether help with decoding was provided (which increased comprehension), and if the text was read aloud or not (silent reading increased comprehension).

## Measuring word reading in text

A primary goal of this study is to examine which lexical and sub-lexical properties of words in a text may make those words more difficult to decode and thus make the text more difficult to read. As noted by Garcia and Cain (2014), the genre of texts can influence text comprehension such that narrative texts are easier to read than expository texts. This finding has been supported in other studies demonstrating that narrative texts are judged to be easier to read than expository texts (Crossley et al., 2023) and may have differing cognitive requirements (Eason et al., 2013; Wu et al., 2020). This difference is likely explained by the differences in lexical features found in words between the genres. For instance, narrative texts often include more high-frequency words and expository texts include more specialized words that are academic in nature (Gardner, 2004) and therefore may require more reliance on decoding skills to read them. Examining the lexical and sub-lexical properties of texts affords opportunities to understand relationships between word reading and reading comprehension and the degree to which ease of reading words can lead to increased comprehension. There are multiple ways to measure the lexical properties of words. A main distinction in these properties is whether the properties are calculated at the lexical or sub-lexical level (Saha et al., 2021).

At the lexical level, the most common approach to measuring word decoding relates to word frequency. Word frequency has been shown to be a strong predictor of text processing and word recognition (Balota & Chumbley, 1984; Crossley et al., 2023; Rayner & Duffy, 1986) and to strongly influence word reading (Joseph et al., 2013; Metsala, 1997). Research indicates that more frequent words are processed and identified more quickly than low-frequency words because of repeated exposure that helps reader accumulate knowledge about a word (Coltheart, Rastle et al., 2001; Reichle et al., 1998). In terms of word recognition, frequent exposure to a word allows readers to recognize it in the absence of spelling-sound correspondences (Gough & Tunmer, 1986). Frequency is also related to contextual diversity (also called range and dispersion). While frequency is the number of times a word occurs in a corpus, contextual diversity is the percentage of documents in a corpus in which a word occurs. Research has indicated that contextual diversity explains psycholinguistic performance better than lexical frequency (Adelman et al., 2006; Baayen, 2012; McDonald and Shillcock 2001). Specifically, these studies find that words that are present in fewer contexts (i.e., more constrained linguistic contexts) take longer to process. Crossley et al. (2023) also reported that reading excerpts that contained more words found in a greater number of contexts led to easier text processing.

Sub-lexical properties of words more accurately relate to decoding as posited in the simple view of reading because they represent grapheme and phoneme correspondences. At the simplest level, counts of graphemes, phonemes, and syllables (and the length of graphemes and syllables) can be calculated to better understand the underlying structures of words. These counts can then be combined to examine letter-to-sound discrepancies, which is a critical aspect of decoding that relates to orthographic transparency depth (Saha et al., 2021). Discrepancy counts for words can be calculated by subtracting the number of phonemes from the number of graphemes (Saha et al., 2021). Counts of graphemes and phonemes can also be used to calculate the number of blends (or clusters of graphemes). Blends are constant clusters that represent multiple phonemes and are difficult for new and struggling readers to learn (Bruck & Treiman, 1990; Treiman, 1991). An example would be the ‘bl’ in ‘blend’. Digraphs, which are pairs of graphemes that represent a single sound (i.e., the ‘th’ in think), are also important in decoding (Henry, 1989).

Related to letter sound discrepancies and blends are counts related to grapheme–phoneme complexity. A deep orthography, like English, has single and multiple letters that correspond to different sounds. Languages with shallow orthographies – where a single letter corresponds to a single sound – are easier to learn to read, whereas languages with deep orthographies lead to more difficult acquisition of reading skills (Frith, Wimmer, & Landerl, 1998). This is because complex grapheme–phoneme relationships, where a single grapheme or grapheme cluster can be pronounced in multiple ways, can cause difficulties for children learning to read (Steacy et al., 2019; Treiman et al., 2006). For instance, the grapheme ‘c’ can be realized as a ‘k’ in the word ‘cat’ and ‘s’ in the word ‘city and the grapheme cluster ‘ss’ can be realized as a ‘s’ sound in ‘boss’, a ‘z’ sound in ‘dessert’, and a ‘sh’ sound in ‘pressure’. Studies have indicated that readers with proficient decoding skills use their knowledge of grapheme–phoneme relationships to read new words which increases reading comprehension and fluency (Eldredge, 2005; Kuhn & Stahl, 2003). Grapheme–phoneme relationships can be calculated based on the probability that a consonant or vowel grapheme will correspond with a particular phoneme, which has been shown to predict the accuracy of word reading (Saha et al., 2021).

Sub-lexical properties of words can also be calculated using measures that tap into phonological awareness. Phonological awareness is the understanding and ability to use and reflect on the sound structures in a language to assist in the processing of that language (Stuart, 2005; Wagner, 1988) and is an important part of lexical restructuring, where words representations in the mental lexicon change over time. (Metsala & Walley, 1998). Phonological awareness is particularly important for languages that do not have a one-to-one mapping between graphemes and phonemes, like English, because when individuals are able to identify individual phonemes in language, it becomes easier for them to learn how to map these individual phonemes onto graphemes. Previous studies have shown strong links between children’s phonological awareness and their later ability to decode words and their vocabulary growth (Hipfner-Boucher et al., 2014; McDowell, Lonigan, & Goldstein, 2007). The phonological structure of words also plays an important role in the development of reading skills (Perfetti & Stafura, 2014; Verhoeven, van Leeuwe, & Vermeer 2011) and is a strong predictor of reading ability (Hulme, 2002; Melby-Lervåg, Lyster, & Hulme, 2012; Pfost, 2015).

Two important features related to phonological awareness that can be calculated are rhymes and neighborhood effects. Research into rhymes indicates that rhyme awareness in children is predictive of reading achievement in later years (Blachman, 1984; Bradley & Bryant, 1983) and aids in the acquisition of both spelling and reading skills (Ball & Blachman, 1991; Lundberg, Frost, & Peterson, 1988; Bryant et al., 1990). Another approach to calculating phonological awareness is through neighborhood density effects. Neighborhood density effects examine how many words can be created from a single word by changing one character (in the case of orthographic neighbors), one phoneme (in the case of phonological neighbors), and one letter and phoneme where the correspondence between the two is exact (in the case of phonographic neighbors). Research has reported that words with denser orthographic and phonologic neighborhoods (i.e., words with a lot of neighbors) are processed more quickly (Crossley et al., 2017; Skalicky, Crossley, & Berger, 2019; Yarkoni et al., 2008).

## Prior measures of word-reading difficulty

Prior research has operationalized word reading difficulty at the lexical and sub-lexical levels in efforts to measure text comprehension. The earliest work in this area was in the development of readability measures that included relatively simple and indirect proxies of decoding, such as the number of letters per word and the number of syllables per word as found in both the Flesch Reading Ease (Flesch, 1948) and the Flesch–Kincaid Grade Level formulas (Kincaid et al., 1975). These measures tap into the notion that the more graphemes or syllables a word has, the more difficult it will be to read. Other readability metrics have used frequency counts to identify words that may be more difficult to read. For example, the New Dale Chall formula (Chall & Dale, 1995) includes counts for the three thousand most common words in English, and more advanced readability models include frequency norms from large corpora (Crossley, 2025; Crossley et al., 2019; Saha et al., 2021; Sheehan, 2017). Indeed, research indicates that frequency norms are significant predictors of text readability. For instance, Crossley et al. (2019) found that texts judged to be more readable included more frequent words, although the frequency metrics were weaker predictors than many other linguistic features.

Recent work has focused on linking readability to more precise and direct measures of decoding at the sub-lexical level. Saha et al. (2021) developed a decoding measure (DM) that included a word frequency metric in addition to metrics related to the number of blends in a word, the conditional probability of vowel and consonant grapheme–phoneme correspondences, and the absolute value of number of letters of words less phonemes in the words. They reported that their DM predicted children’s miscues while reading passages aloud when controlling for age and reading ability.

Crossley and Choi (2024) calculated sub-lexical features related to rhymes and neighborhood effects and used them to model reading comprehension and lexical decision tasks while controlling for word frequency. They reported that the strongest predictors of human judgments of text comprehension were word frequency and the number of rhymes that a word had for the most frequent 1000 words in English. When predicting lexical decision times, which are a measure of decoding, Crossley and Choi found that the number of rhymes words had for the most frequent 1000 and 2500 words in English were strong predictors. Additionally, the number of phonological neighbors a word had, the number of phonographic neighbors, and word frequency were also significant predictors of lexical decision times.

## Tool for Automatic Analysis of Decoding Ambiguity (TAADA) 

The decoding measure reported in Saha et al. (2021) is likely the first such measure created for English. As noted by Saha et al., their decoding measure was not exhaustively refined, and they indicated that additional metrics were needed to reliably measure a word’s decoding difficulty. Specifically, the decoding measure developed by Saha et al. did not include decoding metrics related to word rhymes, neighbor density factors, contextual diversity counts, and other counts related to graphemes, phonemes, and syllables. Lastly, the decoding measure developed by Saha et al. is not easily usable since their online calculator has not been made publicly available to facilitate its use.

**Fig. 1:**
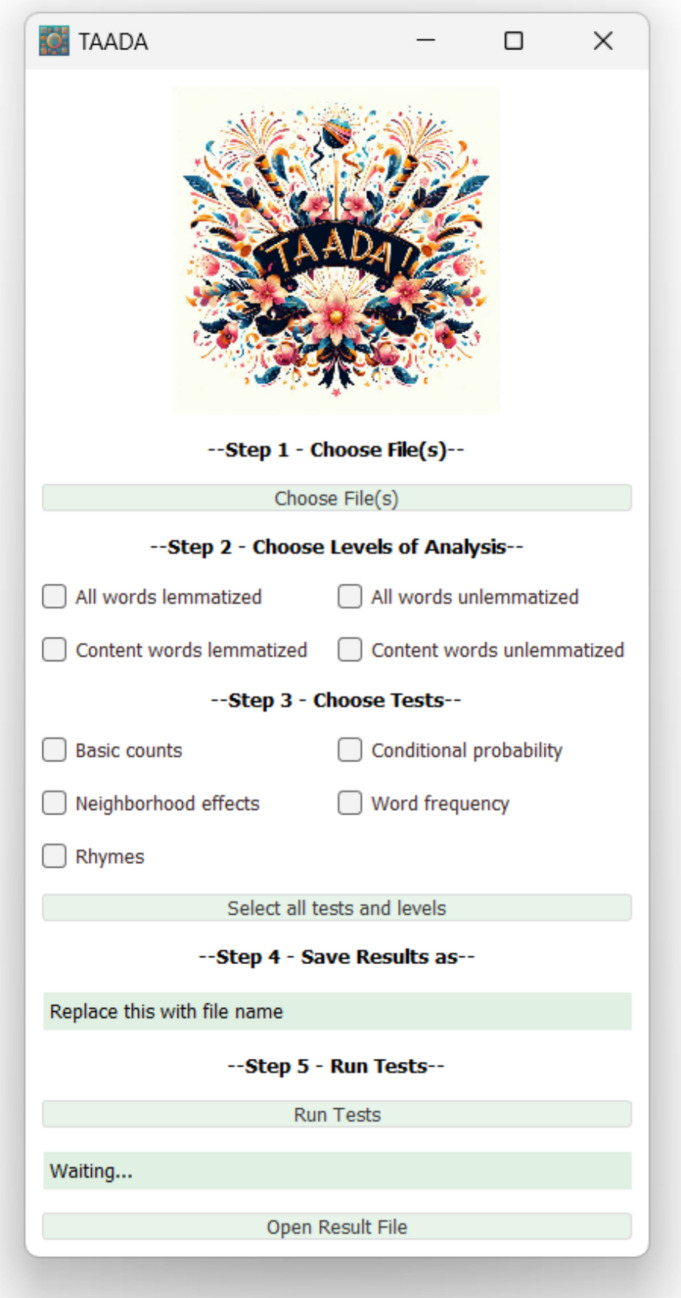
TAADA Interface

In this study, we introduce and assess a decoding tool that builds on the work of Saha et al. (2021): the Tool for the Automatic Analysis of Decoding Ambiguity (TAADA). TAADA is a freely available natural language processing (NLP) tool specifically designed to annotate and count lexical and sub-lexical features related to decoding in English. The features include metrics for grapheme, phoneme, and syllable counts, word frequency, contextual diversity, neighborhood effects, rhymes, and conditional probability. The tool is available at https://www.linguisticanalysistools.org/taada.html and the source code for the tool is available at https://github.com/scrosseye/TAADA. The downloadable version of TAADA is available for both Mac and Windows operating systems and features a graphical user interface (GUI) that allows users to batch process texts (see Fig. 1).

Unlike Saha et al. (2021), TAADA calculates 59 word decoding features as compared to a single measure. Many of the features are derived from available databases and dictionaries, including the CMU pronouncing dictionary (CMUDict), the Berndt et al. (1987) grapheme-to-phoneme correspondence dictionary, the English Lexicon Project database (ELP, Balota et al., 2007), and the Perfect Rhymes dictionary (PeRDict, Crossley & Choi, 2024). All features grouped by decoding categories reported by TAADA are discussed below.

### Basic decoding counts

TAADA includes a number of basic decoding counts derived from the CMU pronouncing dictionary. The CMUdict provides a mapping between a word and that word’s phonemes for 133,779 words in the English language. However, in practice, many of these words are extremely rare.

Prior to use, the CMUdict was cleaned of features that would influence decoding counts. These include removing alternative pronunciations for words, the characters *‘* and from words, and removing any redundant words. In terms of alternative pronunciations, some words in the CMUdict have multiple possible pronunciations. For instance, “abstain” can be pronounced either/æbˈsteɪn/or/əbˈsteɪn/with the former being more common in the United States. The less common pronunciations in the CMUdict are followed by an (*n*) where *n* is the number (up to 3) of a less common pronunciation. For TAADA, we only kept the most common pronunciation. For instance, in the case of “abstain”, the entry for “abstain” was kept and the entry for “abstain(1)” was removed.

After cleaning, the CMUdict included 121,753 words. For these remaining words, we wrote a Python script to calculate the number of letters per word, the number of phonemes per word, and the number of syllables per word (by counting the number of vowel phonemes in each word). We calculated the average length of syllables by dividing the number of letters in a word by the number of syllables. We calculated a raw discrepancy count by subtracting the number of phonemes by the number of letters for each word and a discrepancy ratio by dividing the number of phonemes by the number of letters for each word. We also calculated the number of consonant characters, vowel characters, consonant phonemes, and consonant vowels per word. Lastly, from these counts, we calculated the average phonemes per consonant characters, vowel characters, and all characters per word.

### Word frequency and contextual diversity counts

From the ELP database, we extracted SUBTLEXus frequency counts and contextual diversity (i.e., the number of samples in which a word occurs) counts for each of the words in the CMUDict that overlapped (~50,000 words) with the ELP database. SUBTLEXus is a corpus of subtitles from 8388 American films and television shows that comprise 51 million words (Brysbaert & New, 2009). We specifically selected word frequency and contextual diversity scores from SUBTLEXus that were normed by 1,000,000 words and then logarithmically transformed (log10) to control for Zipfian distributions common to frequency measures (Zipf, 1946). We also derived frequency and contextual diversity scores from the Corpus of Contemporary American English (COCA, Davies, 2009). COCA contains more than one billion words from texts produced from 1990–2019 and includes eight genres: subtitles, spoken, fiction, magazine, web pages, blogs, newspapers, and academic texts. We focused on frequency scores from the magazine genre because the samples were most representative of average language use. Like the SUBTLEXus scores, the COCA values were normed by 1,000,000 words and then logarithmically transformed. COCA frequency and contextual diversity scores were available for ~65,000 of the words in the CMUDict.

### Neighborhood effect counts

TAADA includes word neighborhood measures reported in the English Lexicon Project (ELP) database. The ELP database includes counts for orthographic, phonologic, and phonographic neighbors and their frequency. Neighborhood counts are available for ~45,000 words in the ELP while neighborhood frequency counts are available for between ~22,000 and ~38,000 words depending on the measure.

#### Orthographic neighbors

The ELP database contains measures related to orthographic neighborhood effects for individual words (with and without homonyms). The simplest measure is based on Coltheart’s N (i.e., OrthoN; Coltheart et al., 1979), which is the number of alternate words that can be generated by changing a single letter in a word. For example, the word *cat* includes the orthographic neighbors *oat, cot, vat, cab, mat, cam, bat, rat, cad, hat, cap, pat, fat, sat, eat, car, cut*, and *can* among others (Balota et al., 2007).

The ELP database also reports the mean frequency of the orthographic neighbors per individual words (FreqO) and includes orthographic similarity effects based on Levenshtein distance (Levenshtein, 1966) per word. The measure, called orthographic Levenshtein distance 20 (OLD20, Yarkoni, Balota, & Yap, 2008) measures the average Levenshtein distance between one word and its 20 closest neighbors, where Levenshtein distance quantifies the distance between two words based on the minimum number of substitution, insertion, or deletion operations needed to change one word into another.

#### Phonological neighbors

The ELP database also contains phonological neighborhood features based on phonemes. The ELP calculates the same features for phonological neighbors as it does for orthographic neighbors. These include measures based on Coltheart’s N (i.e., PhonoN), the mean frequency of the phonological neighbors per individual word (FreqP), and phonological neighbors based on Levenshtein distance (PLD). Counts are provided for all words and words that are not homonyms.

##### Rhyme counts

TAADA provides rhyme counts for each word in the Perfect Rhymes Dictionary (PeRDict; Crossley & Choi, 2024). PeRDict includes perfect rhymes (~48,000) based on rhyme patterns extracted from the CMUdict. Because many of the words in the CMUdict are rare and unlikely to influence decoding, PeRDict also provides perfect rhymes for the 1000, 2500, 5000, and 10,000 most frequent words in the English language as identified by COCA.Rhyme counts

##### Conditional probability counts

TAADA includes conditional probability counts for most words in the English language. The conditional probability counts come from Berndt et al. (1987), who calculated grapheme to phoneme/diphthong correspondences for all graphemes and grapheme clusters in English based on a 17,310 corpus of words. To do this, they identified all correspondences between a grapheme (or grapheme cluster) and a phoneme. They then added the frequencies of all these correspondences to derive a total frequency for each grapheme. Lastly, they calculated a reverse probability by dividing the frequency of each individual correspondences by the total frequency for the grapheme.

In practice, the grapheme cluster *OY-E* as found in *gargoyle* has a one-to-one correspondence with the diphthong ɔɪ as does the grapheme cluster *PP* as found *apple* so the reverse probability would be 1. In contrast, the grapheme *I* has six different phonemic realization in English: ɪ as found in *in* (which has a reverse probability of.716), ə as found in civil (which has a reverse probability of.18), aɪ as found in *find* (which has a reverse probability of.074), ɜ as found in *affirm* (which has a reverse probability of.015), j as found in *senior* (which has a reverse probability of.008), and i as in *ski* (which has a reverse probability of.005). Berndt et al. also calculated the prior probabilities of graphemes by dividing the total frequency of a grapheme by the total number of occurrences of all graphemes within their word corpus.

From these counts, we used a Python script to calculate conditional probability counts for English words. The script used regular expressions to identify graphemes and grapheme clusters in words and then calculated probability counts for that word based on the minimum, maximum, and average reverse probability counts for each grapheme or grapheme cluster. In the case of the grapheme *I* above, the maximum count would be.716, the minimum count would be.005, and the mean count would be the average of all six possible phoneme/diphthong conditional probability counts. Weighted counts are also calculated by dividing the min, max, and mean scores by the number of phonemes in the word. Counts were calculated to vowels, consonants, and vowel + consonants.

Additionally, the script calculated the conditional probability counts for each word to derive their conditional probability. We first converted the phonemes in the Berndt dictionary into phonemes used in the CMU (e.g., *ay* to *EY1*, and *ih* to *IH0*, where the numerals demark primary/secondary or absence of stress). After finding some discrepancies between the CMU dictionary and the Berndt data (e.g., The phoneme *IH2* is only represented as the grapheme cluster Y-E in CMU, while it can denote the grapheme cluster EA in Berndt’s data), we elected to remove the stress markers in the CMU dictionary to account for discrepancies. The script then mapped graphemes to phonemes using regular expressions, deriving conditional probability counts for each word. For example, the word *tap* can be broken down into three phonemes *t*, *ae*, and *p*, where the conditional probability of the grapheme *T* mapping to the phoneme *t* is.97 (i.e., P(*t* | *T*) =.97), and P(*ae* | *A*).54, and P(*p | P) =* 1.00. In this example, the average conditional probability (the sum of the conditional probabilities divided by the number of graphemes) for the word *tap* is 0.837. This measure serves as the average predictability of the grapheme and phoneme pairs in a word.

##### TAADA text processing

The TAADA graphical user interface (GUI) reads in plain text files in UTF-8 (Unicode Transformation Format) and extracts decoding statistics for each text based on the calculations outlined above. All texts are first pre-processed using spaCy’s small, English web-based model (Honnibal & Montani, 2017). spaCy is used to tokenize the texts to extract the words. Next, punctuations and stop words from spaCy’s punctuation and stop word list are removed. Stop words were removed because they are frequent words that are often not processed phonologically (Gough & Tunmer, 1986). The stop word list includes 326 stop words and contractions. Stop words include pronouns, copula verbs, conjunctions and connectives, prepositions, some adverbs like *mostly, formerly,* and *really,* and delexical verbs like *give, have, done,* and *be*. Contractions comprise seven items which are *‘ll, ‘re, ‘d, ‘m, ‘s, ‘ve* and *n’t*. Lastly, only alphabetic-numeric tokens are kept.

**Fig. 2:**
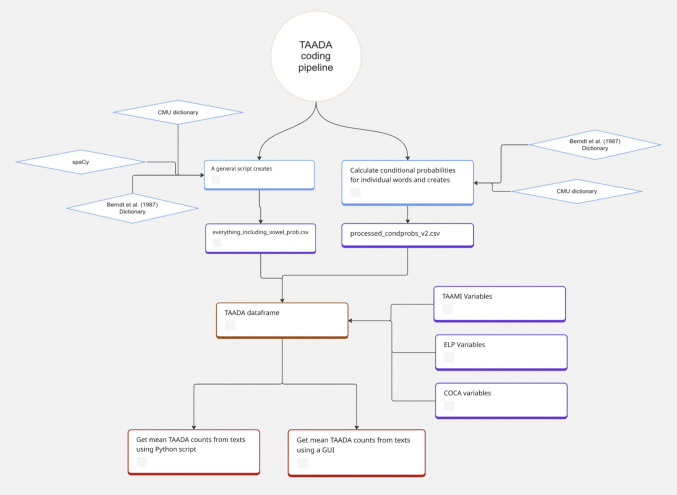
TAADA coding pipeline

Counts are then calculated for all decoding features for all words or for content words only. The TAADA GUI also allows for the extraction of counts only for all lemmas and content lemmas. The output of the TAADA GUI is a comma-separated values (CSV) file that contains the file name and the calculations for all the selected decoding measures averaged across the words in the file. The entire pipeline is visualized in Fig. 2.

## Current studies

We report on two assessments of TAADA that provide validation for the tool. The first study assesses the strength of TAADA indices to predict text readability holistically. Because the readability of a text can be influenced by the decoding difficulty of words contained within a text (Nagy et al., 2006), texts that contain a greater number of words that are more difficult to decode should be judged to be more difficult to comprehend. The second study is a more fine-grained analysis of decoding that examines the strength of TAADA indices to predict whether a word is read aloud correctly or not (i.e., the word leads to miscue). Words that are easier to decode should lead to fewer miscues because they are easier to process.

## Study 1

Our first study is corpus-based and relies on a large-scale collection of text excerpts which each have associated human judgments of readability. The goal of the study is to assess relationships between the TAADA decoding features and the readability judgments.

### Methods

#### Corpus

To assess the links between morphological elements of texts and text readability, we used the CommonLit Ease of Readability (CLEAR) corpus (Crossley et al., 2023). The corpus consists of 4724 text excerpts, which comprise around 800,000 words. The corpus was created for the purpose of developing and evaluating different readability formulas. To obtain distinct readability assessments for the text excerpts, teachers were recruited from CommonLit’s teacher network. Teachers were asked to review pairs of text samples and judge which excerpt was simpler for students to understand. Following the exclusion of outliers, contributions from 1116 educators were retained, amounting to 111,347 comparative assessments in total. A Bradley–Terry model (Bradley & Terry, 1952) was used to compute pairwise comparison scores for the teachers’ judgments of text ease to calculate unique readability scores for each excerpt. The final scores reflect the “Easiness” in terms of comprehension for each excerpt in the corpus. The scoring method and the extraction of readability scores is discussed in Crossley et al. (2023).

#### Statistical analyses

To predict text reading ease scores found in the CLEAR corpus, we used the decoding indices in TAADA as predictor variables in a linear model. We first ensured that none of the TAADA variables correlated strongly with text length (*r* >.699) and also calculated bivariate Pearson correlations for all TAADA variables using the cor.test() function in R (R Core Team, 2021) to identify highly collinear features among the TAADA variables. If two or more variables correlated at *r* >.699, the variable(s) with the lowest correlation with the ease of readability score was removed and the variable with the higher correlation was retained. We also only retained variables that demonstrated at least a small relationship with the ease of readability scores (*r* >.099).

We used the CARET package (Kuhn, 2008) in R to develop linear models. Model training and evaluation were performed using a tenfold cross-validation model using stepwise selection from the leapSeq() function. Estimates of accuracy are reported using the amount of variance explained by the developed models (*R*^2^). The model was checked for suppression effects. The relative importance of the indices in each model was calculated using the calc.relimp() function in the relaimpo package (Grömping, 2006) using the lmg metric (Lindeman et al., 1980). lmg takes into account both the direct relationship between the independent and dependent variable (i.e., the bivariate correlation) and the indirect relationship between the independent and dependent variable (i.e., the amount of variance explained when included in a multivariate model).^1^

### Results

#### Correlations

All variables correlated at *r* <.010 with text length, so no variables were initially removed. Of the 59 variables in TAADA, 44 variables were removed because of multi-collinearity or because they did not report at least a small effect size with the readability score. Correlations among the remaining variables indicated small to medium relationships (*r* <.10 and >.50). Correlations are reported in Table 1.

**Table 1:** Correlations between TAADA variables and text readability

Variable	*r*	*p*
Word frequency (SUBTLEXus)	0.573	<.001
Phonographic neighbors (no homophones)	0.432	<.001
Syllable length	0.351	<.001
Reverse prior probability (all)	– 0.348	<.001
Number rhymes (1000 most frequent words)	0.305	<.001
Phonemes per characters	0.291	<.001
Reverse prior probability (consonants)	–0.276	<.001
Max conditional probability (vowels)	0.255	<.001
Frequency orthographic neighbors	0.207	<.001
Conditional probability (average)	0.204	<.001
Frequency phonological neighbors	0.185	<.001
Phonemes per consonant	0.129	<.001
Number rhymes (10,000 most frequent words)	0.119	<.001
Frequency phonographic neighbors	0.102	<.001

#### Linear model

The 14 variables that remained after controlling for multi-collinearity and for a weak relationship with reading ease scores were entered into a linear model. The ten-fold cross validation model using stepwise selection and controlling for suppression effects included four variables. The model reported *r* =.583, *R*^2^ =.339, *F* (4, 4719) = 606.3, *p* <.001 (see model parameters summarized in Table 2). The relative importance metrics indicate that the strongest predictor of reading ease was related to word frequency followed by phonographic neighbors, syllable length, and the reverse prior probability of consonants. Post hoc tests indicated all linear model assumptions were met.

**Table 2:** Linear model to predict reading ease score using TAADA features

Variable	Relative Importance	Estimate	SE	*t*	*p*
(Intercept)		– 6.965	0.338	– 20.623	<.001
Word frequency (SUBTLEXus)	0.602	1.549	0.051	30.465	<.001
Phonographic neighbors (no homophones)	0.206	0.038	0.014	2.63	<.001
Syllable length	0.123	0.364	0.062	5.924	<.001
Reverse prior probability (consonants)	0.069	– 8.596	4.578	– 1.878	<.001

### Discussion

Our first study examined the potential for decoding features reported by TAADA to predict ease of comprehension in the CLEAR corpus. The reported correlations indicate that TAADA features yielded strong to weak relationships with text readability. Specifically, text readability was strongly related to the frequency of words and moderately related to the number of phonographic neighbors in words, syllable length for words, reverse prior probability for words, and the number of rhymes with very frequent words. Weak relationships were reported for the number of phonemes per characters in words, the reverse prior probability for consonants in words, the max and average conditional probability for words, the frequency of orthographic, phonological and phonographic neighbors in words, phonemes per consonants in words, and the number of rhymes with less frequent words.

These variables were entered into a regression analysis to predict text readability. Four variables were significant predictors of text readability including word frequency, phonographic neighbors for words, word syllable length, and the reverse prior probability for consonants, and these four variables explained 34% of the variance in text readability judgments. The regression coefficients indicated that texts were judged to be easier to read if they contained more frequent words, words that had a greater number of phonographic neighbors, and if they contained words with longer syllables. Additionally, a text was judged easier to read if the probabilities with which a particular grapheme is realized as a particular phoneme in a word is more predictable.

The findings from the first study provide evidence that TAADA variables related to decoding are strong predictors of human judgments of text readability, providing validation for the features. However, the readability outcome variable was operationalized as teacher judgments of which texts would be easiest to read for their students. The outcome variable favors a holistic perspective of readability of which decoding may only be a small part and other elements of reading comprehension such as syntactic complexity, text coherence, or meaning construction may better inform the judgments. Additionally, the judgments are made by adults, who may not strongly depend on decoding to understand a text. Last, the reading judgments are not based on behavioral data and may thus only be a proxy for actual reading.

## Study 2

While our first study examined subjective judgments of readability, our second study analyzes more objective measurements of decoding. The data comes from younger readers tasked with reading a text aloud. The read alouds were recorded and each word produced by the students was coded as being accurate or inaccurately pronounced (i.e., miscues). Miscues were coded as words where a child made a deletion, omission, mispronunciation, substitution or self-correction when reading.

### Methods

#### Dataset

The full dataset used in the second study was extracted from three different read aloud studies conducted in the same lab using the same protocols. Across all three studies, there were 296 total participants who produced 653 distinct words comprising 534 distinct lemmas. In total, there were 120,822 observations of students reading a word aloud, of which participants had no miscues for 114,374 observations and had a miscue for 6475 observations.

The goal of the first study was to identify behavioral and neuronal weaknesses in children that have late-emerging reading difficulties. There were 77 participants in this dataset, who were between the ages of 6 and 8 (M = 7.51, SD = 0.32) when data was collected. The sample was 45.45% male, and their reported race was 62.34% White, 31.17% Black/African American, 5.19% more than one race, and 1.30% Native American. Four passages in this study were taken from the Qualitative Reading Inventory (QRI; Caldwell & Leslie, 2000), which tasked participants with reading passages aloud and answering questions related to the passages to measure word identification, fluency, and comprehension. There were two expository and two narrative passages in total, although participants were counterbalanced to read one narrative and one expository passage out of the four. The first narrative passage was about a mouse in a house, which contained 250 words, and the second was about a surprise gift which contained 210 words. The first expository passage contained 76 words and was about the brain and the five senses. The second was about air and contained 85 words.

The goal of the second study was to understand the role executive functioning plays across reading comprehension development. There were 77 participants in this dataset who were between the ages of 7–9 (M = 8.39, SD = 0.34). The sample was 41.56% male students and reported their race as: 81.82% White, 12.99% Black/African American, 5.20% Asian, 3.90% more than one race, and 2.60% preferred not to answer. These four passages were experimenter-created and controlled to be as identical as possible based on number of words, sentence length, word length, word frequency, word concreteness, reading ease, and grade level (Del Tufo, Earle, & Cutting al., 2019). There were two expository and two narrative passages in total, although participants were counterbalanced to read one narrative and one expository passage out of the four. The first expository passage was about the artic circle, and the second was about hot air balloons. The first narrative passage was about a grasshopper, and the second was about a monkey and a cat. All four passages contained 350 words.

The goal of the third study was to understand fundamental characteristics of the learner in relation to the text complexity features required for skilled reading comprehension. There were 142 participants in this dataset, who were between the ages of 10 and 14 (M = 11.78, SD = 1.34). The sample was 54.23% male students, and the reported races were 76.26% White, 12.95% Black/African American, 2.16% Asian, 5.76% more than one race, and 2.88% preferred not to answer. These two expository passages were experimenter-created and were controlled to be as identical as possible based on cohesion, vocabulary, decoding, and syntax (Spencer et al., 2019). The first expository passage was about deserts, and the second was about toads. Both passages contained 305 words. At the time of analysis, the results from the ‘deserts’ passage had not been coded and, thus, were not included.

#### Data compilation

From the ~120,000 words, we removed all function words so that only content words remained (398 unique words and 54,943 observations). From there, each TAADA feature for each unique word was added to a table. Each of the unique words in the table was checked to see if there were any NA values for the TAADA variables. The variables related to weighted probability counts (min, average, and max) for consonant, vowel, and all character counts showed NA values for three words (*eye, moonless,* and *seethrough*). These words were removed from the data frame leaving 395 unique words and 54,465 observations. We also looked for TAADA variables that showed high zero counts of over 20% of the data (i.e., no reported value for the word). Five TAADA variables showed high zero counts. All variables were related to neighborhood effects (e.g., number of orthographic and phonographic neighbors along with frequency of phonographic and orthographic neighbors) and were removed. The final table included 395 unique words, 54 decoding variables, and 54,465 observations.

The final data frame had a few important considerations. The first is that the data is repeated with each participant providing multiple data points often across more than one passage. The second is that the table showed a strong ceiling effect with 51,287 of the observations showing no miscues and 3178 observations showing miscues. Lastly, the outcome variable in the table was binary (miscue or no miscue).

One concern is that machine learning models trained on the raw data will likely capitalize on the significant imbalanced classification codes reported in the dataset and learn the patterns in the majority class (no miscues) at the expense of the minority class (miscues) because of the distributive properties of the data set. The result would be classification metrics such as precision, recall, and F1 scores that were biased toward the majority class (He & Garcia, 2009). Taking this into consideration, we conducted two sets of analyses. The first was based on an oversampled dataset that randomly samples observations from the minority class in order to balance samples across the two classes. The second was based on the original imbalanced dataset. We selected oversampling versus undersampling because undersampling may cause the classifier to miss important concepts within the over-represented category and because research indicates that oversampling does not suffer from overfitting or other performance degrading effects to the same degree as undersampling (Vanhoeyveld & Martens, 2018). We oversampled using the random over-sampling examples (ROSE; Lunardon et al., 2014) package in R so that we had 51,287 observations for both miscues and non-miscues.

#### Statistical analysis

To examine differences in miscued and correctly spoken words based on decoding variables reported in TAADA, we first controlled for multi-collinearity and effect sizes within the data as reported in Study 1 using dummy coded variables for miscued and correctly spoken words. We then scaled the remaining variables and constructed a generalized linear mixed model (GLMM) using the lme4 package in R. A GLMM can include both fixed and random effects along with binomial distributions when developing a classification model (Faraway, 2016). It uses a logit link function to transform probabilities into a log-odds scale in order to model binary outcomes. In our GLMM model, the response variable was each spoken word as a binomial response defined as either correct (coded as 0) or miscued (coded as 1). The fixed effects were the decoding features from TAADA that were not highly correlated. We had two random effects (intercepts). The first controlled for potential variation across participants and the second controlled for potential variation across passages. We ran two sets of GLMMs. The first set focused on the oversampled data and the second set focused on the original data. For the oversampled data, we first created a baseline model that only included random effects. We then developed a full model that included random and fixed effects. All models were checked for suppression effects and hand-pruned if suppression effects were noted. An ANOVA comparison was made between the baseline and full models to examine differences in strength. Co-efficient estimates along with *z* scores and *p* values are reported along with machine learning metrics including precision, recall, F1, and Kappa scores. The second set of GLMMs were based on the original data (i.e., not the oversampled data) using only the meaningful predictor variables from the oversampled GLMM. Like with the oversampled data, we first created a baseline model that only included random effects. We then created a full model that included random and fixed effects. An ANOVA comparison was made between the baseline model and the final model to examine differences in strength. Because of differences in class sizes, no machine learning metrics are reported for this analysis, but co-efficient estimates along with *z* scores and *p* values are reported.

### Results

#### Variable selection

After controlling for multi-collinearity and effect size, 18 TAADA variables remained. Descriptive statistics for these features are provided in Table 3 along with a delta score, which represents the mean difference between no miscue and miscued words.

**Table 3:** Descriptive statistics for TAADA variables in GLMM analysis

Variable	No miscue	Miscue	Delta
Number of phonemes	3.845	4.078	0.232
Reverse prior probability (vowel)	0.036	0.039	0.003
Number of vowel characters	1.881	1.949	0.068
Phonemes per character (consonants)	1.180	1.212	0.032
Minimum probability (consonant)	0.503	0.523	0.020
Minimum probability (weighted)	0.088	0.093	0.006
Number of rhymes (all words)	34.059	36.105	2.046
Phonemes per character	1.267	1.274	0.007
Number of phonemes (all words)	4.119	4.136	0.017
Average syllable length	3.660	3.662	0.002
Maximum probability (consonants)	0.960	0.959	0.001
Number of rhymes (2500 frequent words)	5.220	4.912	0.308
Phonemes per character (vowel)	1.439	1.408	0.030
Reverse prior probability (consonant)	0.038	0.037	0.002
Conditional probability	0.759	0.747	0.013
Phonological neighborhood frequency (homophones excluded)	6.507	6.255	0.252
Phonographic neighbors (with homophones)	5.967	5.585	0.382
Word frequency (COCA magazine)	3.926	3.794	0.132

#### GLMMs oversampled data

We first constructed a baseline random intercept model that only included participants and texts as random intercepts. This model indicated that intercept was a significant predictor of miscues and reported an *R*^2^ of.426, indicating that miscues are strongly predicted by participant and text variance. A model including both fixed and random effects that was pruned to control for suppression effects (see Table 4). The strongest decoding predictor was related to word frequency followed by variables related to phoneme counts, probability counts, rhyme counts, and conditional probability. The GLMM model reported a marginal *R*^2^ of.030 and a conditional *R*^2^ of.474 indicating that the fixed effects explained ~3% of the variance and the fixed and random effects explained ~44% of the variance. The random effect structure indicated greater variance for participants (variance = 2.183, SD = 1.477) than reading passages (variance = 0.596, SD = 0.772). A comparison between the GLMM with only random effects and the GLMM with fixed and random effects indicated that the latter model was significantly stronger (*x*^*2*^ = 3567.9, *p* <.001).

**Table 4:** GLMM TAADA model for miscue data

Variable	Estimate	*SE*	*z*
Intercept	– 0.555	0.282	– 1.966*
Number of phonemes	0.258	0.010	26.641**
Reverse prior probability (vowel)	0.116	0.010	11.909**
Number of rhymes (all words)	0.116	0.010	11.237**
Phonemes per character (vowel)	0.093	0.010	9.332**
Reverse prior probability (consonant)	– 0.053	0.008	– 6.560**
Conditional probability	– 0.069	0.008	– 8.285**
Word frequency (COCA magazine)	– 0.243	0.008	– 29.186**

** *p* <.001, * *p* <.050

The GLMM correctly allocated 76,821 of the 102,574 observations in the data for an accuracy of.749%. The model reported a recall of.751, precision of.745, and an F1 score of.748 (see Table 5 for the confusion matrix). The reported Cohen’s kappa was *k* =.498, indicating a moderate agreement.

**Table 5:** Confusion matrix for GLMM predictions

	Correct	Miscue
Correct	38,596	12,691
Miscue	13,062	38,225

#### GLMMs original data

We first constructed a baseline random intercept model that only included participants and texts as random intercepts. This model indicated that intercept was a significant predictor of miscues and reported an *R*^*2*^ of.324, indicating that miscues are strongly predicted by participant and text variance. A model including both the fixed and random effects from the oversampled model indicated that all seven predictor variables were significant (see Table 6). Like the analysis using the oversampled data, the strongest decoding predictor was related to word frequency followed by variables related to phoneme counts, probability counts, rhyme counts, and conditional probability. The GLMM model reported a marginal *R*^*2*^ of.036 and a conditional *R*^*2*^ of.404 indicating that the fixed effects explained ~4% of the variance and the fixed and random effects explained ~40% of the variance. The random effect structure indicated greater variance for participants (variance = 1.366, SD = 1.1686) than reading passages (variance = 0.671, SD = 0.819). A comparison between the GLMM with only random effects and the GLMM with fixed and random effects indicated that the latter model was significantly stronger (*x*^*2*^ = 600.61, *p* <.001).

**Table 6:** GLMM TAADA model of miscue data for original data

Variable	Estimate	*SE*	*z*
Intercept	– 3.236	0.288	– 11.234***
Number of phonemes	0.219	0.022	9.898***
Reverse prior probability (vowel)	0.120	0.025	4.825***
Number of rhymes (all words)	0.077	0.015	5.058***
Phonemes per character (vowel)	0.064	0.022	2.868**
Reverse prior probability (consonant)	– 0.044	0.020	– 2.143*
Conditional probability	– 0.080	0.021	– 3.782***
Word frequency (COCA magazine)	– 0.293	0.021	– 13.992***

*** = *p* <.001, ** = *p* <.010, * = *p* <.050

### Discussion

Our second study focused on classifying miscued words based on word-level decoding features derived from TAADA. The data was collected from children between the ages of 6 and14 who read passages aloud. In each passage, individual words were annotated for miscues, which served as the outcome variable for the analyses. The goal of the analysis was to examine if word level decoding features were related to the decoding accuracy. The analysis indicated that seven TAADA variables were significant indicators of student decoding miscues and explained about 3% of the variance in miscues with much of the variance explained by random effects (44%), with greater variation reported for participants over passages. The participant random effects that explained miscues were likely related to factors such as age, reading proficiency, or background knowledge. The passage random effects that explained miscues were likely related to the difficulty of the texts, such as how cohesive they were and the strength of associations between words within the texts.

The results indicated that the strongest linguistic predictors of decoding included word frequency, number of phonemes per word and characters, prior probability of vowels and consonants, and the number of rhymes. The strongest predictor was a measure of word frequency, which indicated that more frequent words had fewer miscues. Basic phoneme counts were also strong predictors and revealed that words with more phonemes and more phonemes per character were more likely to be miscues.

The model also indicated that words that had a greater number of rhymes across all words in the PeRDict dataset were more likely to lead to a miscue. This may sound counter-intuitive, but Crossley and Choi (2024) reported positive correlations with text readability for frequent rhyme counts derived from the most frequent 10,000 words, and negative correlations with rhyme counts derived from all words. They argued that full rhyme counts for a language included many rare and infrequent rhymes that may bias the calculation toward measuring more complex language.

The remaining features were related to conditional probability calculations examining phoneme and grapheme correspondences. These features indicated that words that had fewer miscues had higher conditional probabilities, meaning these words contained grapheme–phoneme correspondences that were more typical. Conditional probabilities averaged across all grapheme and phoneme mappings (i.e., regardless of the pronunciation of the word in which the grapheme and phoneme mappings occurred) showed that words were less likely to be miscued if the probabilities were higher for vowels and lower for consonants. This finding indicates that expected vowel mappings lead to more accurate read alouds, but that the reverse is true for consonant mappings.

## Conclusion

The analyses reported in this paper assess the use of the Tool for the Automatic Analysis of Decoding Ambiguity (TAADA) in two studies. TAADA is an open-source application that includes a graphic user interface to allow for quick and efficient processing of texts. TAADA calculates indices at the lexical and sub-lexical levels related to word frequency, contextual diversity, basic grapheme, letter, and syllable counts, neighborhood effects, rhymes, and conditional probabilities. These features were calculated using native speaker norms for American English.

TAADA variables were assessed in two studies examining text comprehension and decoding. The first study investigated links between decoding variables and judgements of reading ease in a corpus of ~5000 reading excerpts, finding that decoding features explained ~34% of the variance in teachers’ judgments of text readability. The predictive features were related to word frequency, neighborhood density, syllable length, and conditional probability counts. The second study examined links between decoding variables and miscue rates for children between the ages of 6 and 14 while reading passages aloud. In this study, TAADA variables explained about 4% of the variance in miscue rates. The leading predictors were related to word frequency, number of phonemes, number of rhymes, conditional probability counts, and discrepancy counts.

The strongest predictors in both analyses were related to word frequency, underscoring the importance that repeated exposures to words have on reading proficiency. Highly frequent words likely have word-specific representations that lessen the need to decode because readers can link spelling directly to pronunciations (Share, 1995). Thus, more frequent words will lead to greater text comprehension and fewer miscues because readers do not need to rely as strongly on spelling-sound correspondences when reading frequent words. A rhyme feature was also predictive of miscue data indicating that words with a greater number of common rhymes were less likely to lead to miscues, underscoring the importance of phonological awareness-linked metrics. Likewise, a neighborhood density feature was also a strong predictor of judgments of text readability, indicating that words that contained a greater number of phonographic neighbors led to a text that was easier to read.

Decoding features related to basic counts of phonemes and syllables were also strong predictors of reading success, for the miscue and ease of reading datasets respectively. The data demonstrates that reading is more difficult when words have a greater number of phonemes and syllables. A number of grapheme–phoneme probability measures were also significant predictors of readability judgments and reading miscues. These features were generally less predictive than other measures of decoding, though. The measures included average reverse probability metrics for both for vowels and consonants. These measures were not specific to the word but rather provided general metrics about the strength of relationships between graphemes (and grapheme clusters) to their related phonemes. The conditional probability metric that computes grapheme and phoneme probability specific to individual words was also a significant predictor for the miscue data. In total, the probability results indicated that words that had stronger probabilities between graphemes and grapheme clusters and related phonemes led to texts that were easier to read and words that had fewer miscues.

TAADA is the first open-source tool to our knowledge that measures lexical and sub-lexical properties of words related to decoding. It builds on Saha et al. (2021) by including additional measures of decoding at the phoneme, grapheme, and syllable level along with decoding counts specific to conditional probability, rhymes, and neighborhood density. The studies reported here indicate that TAADA features are significantly related to both text readability and reading miscues. While TAADA variables were strong predictors of readability, they only explained a marginal, but significant amount of variance (~3%) in the read aloud miscue dataset. Much of the variance in this study was explained by participant effects (~44%) indicating that successful reading aloud is generally a function of individual differences in a reader’s reading proficiency, background knowledge, age, and other factors not included in this study. Additionally, the second study depended on over-sampling the miscue data, which may lead to results that are not generalizable outside of the current study because the natural distribution of the data was likely altered. Oversampling can sometimes lead to overfitting, leading to poor performance on unseen data (He & Garcia, 2009). Thus, while the current study shows support for the use of lexical and sub-lexical features to better understand decoding, the features need to be assessed in other contexts including different tasks, different genres, and different populations. While we presume that lexical and sub-lexical properties of words can help us better understand the impacts of each on reading outcomes, this notion needs to be more thoroughly investigated.

Additionally, while TAADA includes a variety of features related to decoding, it is not an exhaustive list of all features. For instance, the rhyme counts reported in PeRDict (Crossley & Choi, 2024) are only for perfect rhymes and do not include onset rhymes, which may affect word processing (Christensen, 2009). Likewise, the neighborhood density measures reported in the ELP (Balota et al., 2007) make no distinctions for rhyme versus non-rhyme neighbors, which may influence word decoding. Some of the databases TAADA taps into are also dated. For instance, the grapheme to phoneme correspondence dictionary reported by Berndt et al. (1987) is almost 40 years old and designed on a relatively small sample of 17,310 words collected 60 years ago (Hanna et al., 1966). Larger corpora are now available and similar grapheme to phoneme correspondence dictionaries should be developed to better reflect current language analytic knowledge.

Lastly, TAADA only focuses on the English language and has an implicit focus on American English because the norms used for calculating the features are based on dictionaries informed through standard American English. Although all Englishes share strong similarities, the results reported here may differ for data collected in British English, World English, and non-native English settings. Future work could focus on multi-lingual versions of TAADA which could assess how phonological differences influence decoding ability for different language populations. For instance, many languages have orthographic and phonologic mappings that are unambiguous, which are known to lesson demands on decoding skills and therefore likely increase the weight of importance of other lexical features for reading words (i.e., the importance of frequency or syllable length). Developing similar tools to TAADA in other languages would allow researchers to assess theories related to phonological grain size and their effects on decoding across languages (Elbro & Pallesen, 2002; Ziegler & Goswami, 2005).

While future studies and work are needed to improve upon approaches that automatically annotate words for lexical and sub-lexical features of decoding, the current version of TAADA provides researchers with simple and reliable methods to automatically assess many components of decoding. We expect that TAADA can be used in a variety of studies that examine the intersection of decoding, word processing, and text comprehension. TAADA has application beyond reading studies as well because researchers involved in language assessment, language development, language acquisition, and other cognitive modeling areas can benefit from better understanding lexical and sub-lexical word properties.

## Data Availability

All data and materials for all studies reported in this paper except the miscue data are available at https://github.com/scrosseye/TAADA_Analyses. The miscue data cannot be shared according to approved IRB protocols because it was collected from minors.

## Footnotes

1 All R scripts and data for the analyses in this paper are available at https://github.com/scrosseye/TAADA_Analyses

## Code availability

The base code for the TAADA tool can be found at https://github.com/scrosseye/TAADA. The tool is available at https://www.linguisticanalysistools.org/taada.html as a desktop application.

## References

[CR1] Adelman, J. S., Brown, G. D. A., & Quesada, J. F. (2006). Contextual diversity, not word frequency, determined word-naming and lexical decision times. *Psychological Science,**17*, 814–823. 10.1111/j.1467-9280.2006.01787.x16984300 16984300 10.1111/j.1467-9280.2006.01787.x

[CR2] Adelman, J. S., Marquis, S. J., Sabatos-DeVito, M. G., & Estes, Z. (2013). The unexplained nature of reading. *Journal of Experimental Psychology: Learning, Memory, and Cognition,**39*(4), 1037. 23565795 10.1037/a0031829

[CR3] Allington, R. L. (2005). The other five “pillars” of effective reading instruction. *Reading Today,**22*(6), 3.

[CR4] Baayen, H. (2012). Demythologizing the word frequency effect: A discriminative learning perspective. In *Methodological and Analytic Frontiers in Lexical Research* (pp. 171–195). John Benjamins Publishing Company.

[CR5] Ball, E. W., & Blachman, B. A. (1991). Does phoneme awareness training in kindergarten make a difference in early word recognition and developmental spelling? *Reading Research Quarterly*. 10.1598/RRQ.26.1.3

[CR6] Balota, D. A., & Chumbley, J. I. (1984). Are lexical decisions a good measure of lexical access? The role of word frequency in the neglected decision stage. *Journal of Experimental Psychology: Human Perception and Performance,**10*(3), 340. 6242411 10.1037//0096-1523.10.3.340

[CR7] Balota, D. A., Yap, M. J., Cortese, M. J., Hutchison, K. A., Kessler, B., Loftis, B., Neely, J., Nelson, D., Simpson, G., & Treiman, R. (2007). The English lexicon project. *Behavior Research Methods,**39*, 445–459. 10.3758/BF03193014 17958156 10.3758/bf03193014

[CR8] Berninger, V. W., Abbott, R. D., Vermeulen, K., & Fulton, C. M. (2006). Paths to reading comprehension in at-risk second-grade readers. *Journal of Learning Disabilities,**39*(4), 334–351. 16895158 10.1177/00222194060390040701

[CR9] Berndt, R. S., Reggia, J. A., & Mitchum, C. C. (1987). Empirically derived probabilities for grapheme-to-phoneme correspondences in English. *Behavior Research Methods, Instruments, & Computers,**19*(1), 1–9.

[CR10] Best, R. M., Floyd, R. G., & McNamara, D. S. (2008). Differential competencies contributing to children’s comprehension of narrative and expository texts. *Reading Psychology,**29*(2), 137–164.

[CR11] Blachman, B. A. (1984). Relationship of rapid naming ability and language analysis skills to kindergarten and first-grade reading achievement. *Journal of Educational Psychology,**76*(4), 610.

[CR12] Bradley, L., & Bryant, P. E. (1983). Categorizing sounds and learning to read—A causal connection. *Nature,**301*(5899), 419–421.

[CR13] Bradley, R. A., & Terry, M. E. (1952). Rank analysis of incomplete block designs. I. The method of paired comparisons. *Biometrika,**39*, 324–345.

[CR14] Bruck, M., & Treiman, R. (1990). Phonological awareness and spelling in normal children and dyslexics: The case of initial consonant clusters. *Journal of Experimental Child Psychology,**50*(1), 156–178. 2398331 10.1016/0022-0965(90)90037-9

[CR15] Bryant, P. E., MacLean, M., Bradley, L. L., & Crossland, J. (1990). Rhyme and alliteration, phoneme detection, and learning to read. *Developmental Psychology,**26*(3), 429.

[CR16] Brysbaert, M., & New, B. (2009). Moving beyond Kucera and Francis: A critical evaluation of current word frequency norms and the introduction of a new and improved word frequency measure for American English. *Behavior Research Methods,**41*, 977–990. 10.3758/BRM.41.4.977 19897807 10.3758/BRM.41.4.977

[CR17] Caldwell, J., & Leslie, L. (2000). *Qualitative reading inventory* (3rd ed.). Allyn & Bacon.

[CR18] Chall, J. S., & Dale, E. (1995). *Readability revisited: The new Dale-Chall readability formula*. Brookline Books.

[CR19] Cheatham, J. P., & Allor, J. H. (2012). The influence of decodability in early reading text on reading achievement: A review of the evidence. *Reading and Writing,**25*(9), 2223–2246. 10.1007/s11145-011-9355-2

[CR20] Christensen, C. A. (2009). Supporting students with reading difficulties within a whole school approach to literacy. *Australian Journal of Dyslexia and Other Learning Disabilities,**4*, 3–14.

[CR21] Collins-Thompson, K. (2014). Computational assessment of text readability: A survey of current and future research. *ITL-International Journal of Applied Linguistics,**165*(2), 97–135.

[CR22] Coltheart, M., Besner, D., Jonasson, J. T., & Davelaar, E. (1979). Phonological encoding in the lexical decision task. *Quarterly Journal of Experimental Psychology,**31*(3), 489–507.

[CR23] Coltheart, V., Laxon, V. J., & Keating, C. (1988). Effects of word imageability and age of acquisition on children’s reading. *British Journal of Psychology,**79*(1), 1–12.

[CR24] Coltheart, M., Rastle, K., Perry, C., Langdon, R., & Ziegler, J. (2001). DRC: A dual route cascaded model of visual word recognition and reading aloud. *Psychological Review,**108*(1), 204. 11212628 10.1037/0033-295x.108.1.204

[CR25] Crossley, S. A. (2025). Developing linguistic constructs of text readability using natural language processing. *Scientific Studies of Reading,**29*(2), 138–160.

[CR26] Crossley, S. A., & Choi, J. S. (2024). Measuring phonological complexity using the Perfect Rhymes Dictionary (PeRDict). *Reading Psychology,**45*(8), 775–802.

[CR27] Crossley, S. A., Heintz, A., Choi, J., Batchelor, J., Karimi, M., & Malatinszky, A. (2023). A large-scaled corpus for assessing text readability. *Behavior Research Methods,**55*, 491–507. 35297016 10.3758/s13428-022-01802-x PMC10027808

[CR28] Crossley, S. A., Skalicky, S., Dascalu, M., McNamara, D., & Kyle, K. (2017). Predicting text comprehension, processing, and familiarity in adult readers: New approaches to readability formulas. *Discourse Processes,**54*(5–6), 340–359.

[CR29] Crossley, S. A., Skalicky, S., & Dascalu, M. (2019). Moving beyond classic readability formulas: New methods and new models. *Journal of Research in Reading, 42*(3-4), 541–561.

[CR30] Davies, M. (2009). The 385+ million-word corpus of contemporary American English (1990–2008+): Design, architecture, and linguistic insights. *International Journal of Corpus Linguistics,**14*(2), 159–190.

[CR31] Del Tufo, S. N., Earle, S., & Cutting, L. E. (2019). The impact of expressive language development and the left inferior longitudinal fasciculus on listening and reading comprehension. *Journal of Neurodevelopmental Disorders,**11*, Article 37. 10.1186/s11689-019-9296-7 31838999 10.1186/s11689-019-9296-7 PMC6912995

[CR32] Eason, S. H., Sabatini, J. P., Goldberg, L. F., Bruce, K. M. & Cutting, L. E. (2013). Examining the relationship between word reading efficiency and oral reading rate in predicting comprehension among different types of readers. *Scientific Studies of Reading, 17,* 199–223. PMCID: PMC3646383. 10.1080/10888438.2011.652722 PMC3646383 23667307

[CR33] Ehri, L. C. (2014). Orthographic mapping in the acquisition of sight word reading, spelling memory, and vocabulary learning. *Scientific Studies of Reading,**18*(1), 5–21.

[CR34] Ehri, L. C., Nunes, S. R., Stahl, S. A., & Willows, D. M. (2001). Systematic phonics instruction helps students learn to read: Evidence from the National Reading Panel’s meta-analysis. *Review of Educational Research,**71*(3), 393–447.

[CR35] Elbro, C., & Pallesen, B. R. (2002). The quality of phonological representations: A causal link? In L. Verhoeven, C. Elbro, & P. Reitsma (Eds.), *Precursors of Functional Literacy* (Vol. 11, pp. 17–32). John Benjamins.

[CR36] Eldredge, J. L. (2005). Foundations of fluency: An exploration. *Reading Psychology,**26*(2), 161–181.

[CR37] Faraway, J. J. (2016). *Extending the linear model with R: generalized linear, mixed effects and nonparametric regression models*. Chapman and Hall/CRC.

[CR38] Flesch, R. (1948). A new readability yardstick. *Journal of Applied Psychology,**32*, 221–233. 18867058 10.1037/h0057532

[CR39] Frith, U., Wimmer, H., & Landerl, K. (1998). Differences in phonological recoding in German-and English-speaking children. *Scientific Studies of Reading,**2*(1), 31–54.

[CR40] García, J. R., & Cain, K. (2014). Decoding and reading comprehension: A meta-analysis to identify which reader and assessment characteristics influence the strength of the relationship in English. *Review of Educational Research,**84*(1), 74–111.

[CR41] Gardner, D. (2004). Vocabulary input through extensive reading: A comparison of words found in children’s narrative and expository reading materials. *Applied Linguistics,**25*(1), 1–37.

[CR42] Gough, P. B., Hoover, W. A., & Peterson, C. L. (1996). Some observations on a Simple View on Reading. In C. Cornoldi & J. Oakhill (Eds.), *Reading Comprehension Difficulties* (pp. 1–13). LEA.

[CR43] Gough, P. B., & Tunmer, W. E. (1986). Decoding, reading, and reading disability. *Remedial and Special Education,**7*(1), 6–10.

[CR44] Grömping, U. (2006). R package relaimpo: Relative importance for linear regression. *Journal of Statistical Software,**17*(1), 139–147.

[CR45] Hanna, P. R., Hanna, J. S., Bergquist, S. R., Hodges, R. E., & Rudorf, E. H. (1966). Needed research in spelling. *Elementary English,**43*(1), 60–89.

[CR46] He, H., & Garcia, E. A. (2009). Learning from imbalanced data. *IEEE Transactions on Knowledge and Data Engineering,**21*(9), 1263–1284.

[CR47] Henry, M. K. (1989). Children’s word structure knowledge: Implications for decoding and spelling instruction. *Reading and Writing,**1*, 135–152.

[CR48] Hipfner-Boucher, K., Milburn, T., Weitzman, E., Greenberg, J., Pelletier, J., & Girolametto, L. (2014). Relationships between preschoolers’ oral language and phonological awareness. *First Language,**34*(2), 178–197. 10.1044/2015_LSHSS-14-0020 25615430

[CR49] Hoover, W. A., & Gough, P. B. (1990). The simple view of reading. *Reading and Writing,**2*, 127–160.

[CR50] Honnibal, M., & Montani, I. (2017). *spaCy 2: Natural language understanding with Bloom embeddings, convolutional neural networks and incremental parsing*.

[CR51] Howes, D. H., & Solomon, R. L. (1951). Visual duration thresholds as a function of word probability. *Journal of Experimental Psychology,**41*(6), 401–410. 14873866 10.1037/h0056020

[CR52] Hulme, C. (2002). Phonemes, rimes, and the mechanisms of early reading development. *Journal of Experimental Child Psychology,**82*(1), 58–64. 10.1006/jecp.2002.2670 12081455

[CR53] Jenkins, J. R., Fuchs, L. S., Van Den Broek, P., Espin, C., & Deno, S. L. (2003). Sources of individual differences in reading comprehension and reading fluency. *Journal Of Educational Psychology,**95*(4), 719.

[CR54] Johnston, T. C., & Kirby, J. R. (2006). The contribution of naming speed to the simple view of reading. *Reading and Writing,**19*, 339–361.

[CR55] Joseph, H. S., Nation, K., & Liversedge, S. P. (2013). Using eye movements to investigate word frequency effects in children’s sentence reading. *School Psychology Review,**42*(2), 207–222.

[CR56] Juel, C., & Solso, R. L. (1981). The role of orthographic redundancy, versatility and spelling-sound correspondences in word identification. *Directions in Reading: Research and Instruction*, 74–82.

[CR57] Just, M. A., & Carpenter, P. A. (1980). A theory of reading: From eye fixations to comprehension. *Psychological Review,**87*(4), 329. 7413885

[CR58] Just, M. A., & Carpenter, P. A. (1987). *The psychology of reading and language comprehension*. Allyn & Bacon.

[CR59] Katzir, T., Kim, Y., Wolf, M., O’Brien, B., Kennedy, B., Lovett, M., & Morris, R. (2006). Reading fluency: The whole is more than the parts. *Annals of Dyslexia,**56*, 51–82. 17849208 10.1007/s11881-006-0003-5

[CR60] Kern, M. L., & Friedman, H. S. (2009). Early educational milestones as predictors of lifelong academic achievement, midlife adjustment, and longevity. *Journal of Applied Developmental Psychology,**30*(4), 419–430. 10.1016/j.appdev.2008.12.025 PMC2713445 19626128

[CR61] Kincaid, J. P., Fishburne, R. P., Rogers, R. L., & Chissom, B. S. (1975). *Derivation of new readability Formulas: (Automated readability index, fog count and Flesch Reading Ease Formula) for Navy enlisted personnel*. (No. RBR-8-75). Naval Technical Training Command, Millington, TN: Research Branch.

[CR62] Kuhn, M. (2008). Building predictive models in R using the caret package. *Journal of Statistical Software,**28*, 1–26.27774042

[CR63] Kuhn, M. R., & Stahl, S. A. (2003). Fluency: A review of developmental and remedial practices. *Journal of Educational Psychology,**95*(1), 3.

[CR64] Levenshtein, V. I. (1966). Binary codes capable of correcting deletions, insertions, and reversals. *Soviet Physics, Doklady,**10*(8), 707–710.

[CR65] Lindeman, R. H., Merenda, P. F., & Gold, R. Z. (1980). *Introduction to bivariate and multivariate analysis* (Vol. 4). Glenview, IL: Scott, Foresman.

[CR66] Lunardon, N., Menardi, G., & Torelli, N. (2014). ROSE: A package for binary imbalanced learning. *The R Journal*. 10.32614/RJ-2014-008

[CR67] Lundberg, I., Frost, J., & Peterson, O. (1988). Effects of an extensive program for stimulating phonological awareness in preschool children. *Reading Research Quarterly,**23*, 263–284.

[CR68] McDonald, S. A., & Shillcock, R. C. (2001). Rethinking the word frequency effect: The neglected role of distributional information in lexical processing. *Language and Speech,**44*, 295–323. 11814216 10.1177/00238309010440030101

[CR69] McDowell, K. D., Lonigan, C. J., & Goldstein, H. (2007). Relations among socioeconomic status, age, and predictors of phonological awareness. *Journal of Speech, Language, and Hearing Research*. 10.1044/1092-4388(2007/075) 17675606 10.1044/1092-4388(2007/075)

[CR70] McLaughlin, M. J., Speirs, K. E., & Shenassa, E. D. (2014). Reading disability and adult attained education and income: Evidence from a 30-year longitudinal study of a population-based sample. *Journal of Learning Disabilities,**47*(4), 374–386. 22983608 10.1177/0022219412458323

[CR71] Melby-Lervåg, M., Lyster, S. A. H., & Hulme, C. (2012). Phonological skills and their role in learning to read: A meta-analytic review. *Psychological Bulletin,**138*(2), 322. 22250824 10.1037/a0026744

[CR72] Mesmer, H. A. (2005). Decodable text and the first-grade reader. *Reading & Writing Quarterly,**21*(1), 61–86. 10.1080/10573560590523667

[CR73] Mesmer, H. A., Cunningham, J. W., & Elfrieda, H. H. (2012). Toward a theoretical model of text complexity for the early grades: Learning from the past, anticipating the future. *Reading Research Quarterly,**47*(3), 235–258.

[CR74] Metsala, J. L. (1997). An examination of word frequency and neighborhood density in the development of spoken-word recognition. *Memory & Cognition,**25*(1), 47–56. 9046869 10.3758/bf03197284

[CR75] Metsala, J. L., & Walley, A. C. (1998). Spoken vocabulary growth and the segmental restructuring of lexical representations: Precursors to phonemic awareness and early reading ability. In J. L. Metsala & L. C. Ehri (Eds.), *Word Recognition in Beginning Literacy* (pp. 89–120). Erlbaum.

[CR76] Nagy, W., Berninger, V. W., & Abbott, R. D. (2006). Contributions of morphology beyond phonology to literacy outcomes of upper elementary and middle-school students. *Journal of Educational Psychology,**98*(1), 134.

[CR77] Nation, K., Angell, P., & Castles, A. (2007). Orthographic learning via self-teaching in children learning to read English: Effects of exposure, durability, and context. *Journal of Experimental Child Psychology,**96*(1), 71–84. 16904123 10.1016/j.jecp.2006.06.004

[CR78] O’Reilly, T., Sabatini, J., & Wang, Z. (2019). What you don’t know won’t hurt you, unless you don’t know you’re wrong. *Reading Psychology,**40*(7), 638–677.

[CR79] Perfetti, C. A. (1985). *Reading ability*. Oxford University Press.

[CR80] Perfetti, C. A., & Hart, L. (2008). The lexical quality hypothesis. In L. Verhoeven, C. Elbro, & P. Reitsma (Eds.), *Precursors of Functional Literacy* (Vol. 11, pp. 189–213). John Benjamins.

[CR81] Pickren, S. E., Stacy, M., Del Tufo, S. N., Spencer, M., & Cutting, L. E. (2022). The contribution of text characteristics to reading comprehension: Investigating the influence of text emotionality. *Reading Research Quarterly,**57*, 649–667. 35492809 10.1002/rrq.431 PMC9049824

[CR82] Perfetti, C., & Stafura, J. (2014). Word knowledge in a theory of reading comprehension. *Scientific Studies of Reading,**18*(1), 22–37.

[CR83] Pfost, M. (2015). Children’s phonological awareness as a predictor of reading and spelling. *Zeitschrift Für Entwicklungspsychologie Und Pädagogische Psychologie*. 10.1026/0049-8637/a000141

[CR84] R Core Team (2021). R: A language and environment for statistical computing. R Foundation for Statistical Computing, Vienna, Austria. https://www.R-project.org/.

[CR85] Reichle, E. D., Pollatsek, A., Fisher, D. L., & Rayner, K. (1998). Toward a model of eye movement control in reading. *Psychological Review,**105*(1), 125. 9450374 10.1037/0033-295x.105.1.125

[CR86] Rayner, K., & Duffy, S. A. (1986). Lexical complexity and fixation times in reading: Effects of word frequency, verb complexity, and lexical ambiguity. *Memory & Cognition,**14*(3), 191–201. 3736392 10.3758/bf03197692

[CR87] Richardson, J. T. E. (1975). The effect of word imageability in acquired dyslexia. *Neuropsychologia,**13*(3), 281–288. 1161127 10.1016/0028-3932(75)90004-4

[CR88] Saenz, L. M., & Fuchs, L. S. (2002). Examining the reading difficulty of secondary students with learning disabilities: Expository versus narrative text. *Remedial and Special Education,**23*(1), 31–41.

[CR89] Saha, N. M., Cutting, L. E., Del Tufo, S., & Bailey, S. (2021). Initial validation of a measure of decoding difficulty as a unique predictor of miscues and passage reading fluency. *Reading and Writing,**34*, 497–527. 33814724 10.1007/s11145-020-10073-x PMC8011635

[CR90] Savage, R., & Stuart, M. (2006). A developmental model of reading acquisition based upon early scaffolding errors and subsequent vowel inferences. *Educational Psychology,**26*(1), 33–53.

[CR91] Seidenberg, M. S., Farry‐Thorn, M., & Zevin, J. D. (2022). Models of word reading: What have we learned? *The Science of Reading: A Handbook*, 36–59.

[CR92] Share, D. L. (1995). Phonological recoding and self-teaching: Sine qua non of reading acquisition. *Cognition,**55*(2), 151–218. 7789090 10.1016/0010-0277(94)00645-2

[CR93] Sheehan, K. M. (2017). Validating automated measures of text complexity. *Educational Measurement: Issues and Practice,**36*(4), 35–43.

[CR94] Skalicky, S., Crossley, S. A., & Berger, C. M. (2019). Predictors of second language English lexical recognition: Further insights from a large database of second language lexical decision times. *The Mental Lexicon,**14*(3), 333–356.

[CR95] Snow, C. (Ed.). (2002). *Reading for understanding: Toward an R & D program in reading comprehension*. Santa Monica, CA: Rand.

[CR96] Spencer, M., Gilmour, A. F., Miller, A. C., Emerson, A. M., Saha, N. M., & Cutting, L. E. (2019). Understanding the influence of text complexity and question type on reading outcomes. *Reading and Writing,**32*(3), 603–637. 10.1007/s11145-018-9883-0 30983698 10.1007/s11145-018-9883-0 PMC6455959

[CR97] Stanovich, K. E. (1985). Explaining the variance in reading ability in terms of psychological processes: What have we learned? *Annals of Dyslexia,**35*, 67–96. 10.1007/BF02659181 24243410 10.1007/BF02659181

[CR98] Steacy, L. M., Wade-Woolley, L., Rueckl, J. G., Pugh, K. R., Elliott, J. D., & Compton, D. L. (2019). The role of set for variability in irregular word reading: Word and child predictors in typically developing readers and students at-risk for reading disabilities. *Scientific Studies of Reading,**23*(6), 523–532. 32855591 10.1080/10888438.2019.1620749 PMC7449249

[CR99] Stuart, M. (2005). Phonemic analysis and reading development: Some current issues. *Journal of Research in Reading,**28*(1), 39–49.

[CR100] Taylor, J. N., & Perfetti, C. A. (2016). Eye movements reveal readers’ lexical quality and reading experience. *Reading and Writing,**29*, 1069–1103.

[CR101] Tilstra, J., McMaster, K., den Van Broek, P., Kendeou, P., & Rapp, D. (2009). Simple but complex: Components of the simple view of reading across grade levels. *Journal of Research in Reading,**32*(4), 383–401.

[CR102] Treiman, R. (1991). Phonological awareness and its roles in learning to read and spell. In *Phonological awareness in reading: The evolution of current perspectives* (pp. 159–189). New York, NY: Springer New York.

[CR103] Treiman, R., Kessler, B., Zevin, J. D., Bick, S., & Davis, M. (2006). Influence of consonantal context on the reading of vowels: Evidence from children. *Journal of Experimental Child Psychology,**93*(1), 1–24. 16115645 10.1016/j.jecp.2005.06.008

[CR104] U.S. Department of Education. Institute of Education Sciences, National Center for Education Statistics, National Assessment of Educational Progress (NAEP), 2024 Reading Assessment.

[CR105] Vanhoeyveld, J., & Martens, D. (2018). Imbalanced classification in sparse and large behaviour datasets. *Data Mining and Knowledge Discovery,**32*(1), 25–82.

[CR106] Verhoeven, L., van Leeuwe, J., & Vermeer, A. (2011). Vocabulary growth and reading development across the elementary school years. *Scientific Studies of Reading,**15*(1), 8–25.

[CR107] Wagner, R. K. (1988). Causal relations between the development of phonological processing abilities and the acquisition of reading skills: A meta-analysis. *Merrill-Palmer Quarterly (1982–)*, 261–279.

[CR108] Wang, Z., Sabatini, J., O’Reilly, T., & Feng, G. (2017). How individual differences interact with task demands in text processing. *Scientific Studies of Reading,**21*(2), 165–178.

[CR109] Wolfe, M. B., Schreiner, M. E., Rehder, B., Laham, D., Foltz, P. W., Kintsch, W., & Landauer, T. K. (1998). Learning from text: Matching readers and texts by latent semantic analysis. *Discourse Processes,**25*(2–3), 309–336.

[CR110] Wu, Y., Barquero, L.A., Pickren, S., Taboada-Barber, A.M., & Cutting, L.E. (2020). The relation between cognitive skills and reading comprehension of narrative and expository texts: A longitudinal study from Grade 1 to Grade 4. *Learning and Individual Differences, 80.*10.1016/j.lindif.2020.101848 10.1016/j.lindif.2020.101848 PMC7291864 32536780

[CR111] Yarkoni, T., Balota, D., & Yap, M. (2008). Moving beyond Coltheart’s N: A new measure of orthographic similarity. *Psychonomic Bulletin & Review,**15*(5), 971–979. 18926991 10.3758/PBR.15.5.971

[CR112] Ziegler, J. C., & Goswami, U. (2005). Reading acquisition, developmental dyslexia, and skilled reading across languages: A psycholinguistic grain size theory. *Psychological Bulletin,**131*(1), 3. 15631549 10.1037/0033-2909.131.1.3

[CR113] Zipf, G. K. (1946). The psychology of language. In *Encyclopedia of Psychology* (pp. 332–341). Philosophical Library.

